# Dropping Objects in Carpal Tunnel Syndrome: Clinical and Electrophysiological Features

**DOI:** 10.7759/cureus.67504

**Published:** 2024-08-22

**Authors:** Zeliha Matur, Sule Deveci, Emine Taskiran, Ali Emre Oge

**Affiliations:** 1 Neurology, Bezmialem Vakif University, Faculty of Medicine, Istanbul, TUR; 2 Neurology, Başakşehir Çam and Sakura City Hospital, Istanbul, TUR; 3 Neurology, Istanbul University-Cerrahpasa, Cerrahpasa Medical Faculty, Istanbul, TUR; 4 Neurology and Clinical Neurophysiology, Istanbul University, Istanbul Faculty of Medicine, Istanbul, TUR

**Keywords:** median motor conduction velocity, median motor conduction block, boston carpal tunnel syndrome questionnaire, carpal tunnel syndrome, dropping objects

## Abstract

Background

Dysfunction in both afferent sensory and efferent motor components of the median nerve may contribute to the manifestation of the symptom of dropping objects (DO) in carpal tunnel syndrome (CTS). The objective of this study was to compare the clinical and electrophysiological characteristics of CTS patients with dropping objects (wDO) and those without dropping objects (w/oDO). The study evaluated the correlation between DO symptoms and median partial conduction block, as well as the reduction in median motor conduction velocity at the wrist.

Methodology

An examination for CTS and the Boston Carpal Tunnel Syndrome Questionnaire (BCTQ) were performed. Median and ulnar sensory and motor nerve conduction studies were conducted in both upper extremities, and the percentage decrease in the median compound motor action potential amplitude at the wrist level was calculated.

Results

The study included 201 female participants aged between 22 and 88 years, comprising 57 patients with CTSwDO, 78 patients with CTSw/oDO, and 66 healthy controls. In the CTSwDO group, compared to the CTSw/oDO group, BCTQ scores were significantly higher, the prevalence of sensory deficits was more pronounced, and a larger number of hands, both dominant and non-dominant, were classified as stage 3 or higher in the clinical CTS classification. However, no correlation was found between the presence of DO symptoms and any neurophysiological findings.

Conclusions

DO may be a symptom of clinical severity in CTS, as previously reported. No neurophysiological parameter that could be associated with DO was identified.

## Introduction

Carpal tunnel syndrome (CTS) is the most common entrapment neuropathy with a prevalence of approximately 3%, resulting from compression of the median nerve as it passes through the carpal tunnel [1,2]. The most common symptoms of CTS include pain in the wrist, numbness and tingling in the hand, particularly in the first three fingers, difficulty grasping objects or performing activities requiring a strong grip, and numbness, tingling, or pain in the hand that may awaken from sleep at night. In severe cases, paraesthesia may be felt throughout the entire hand and may radiate up to the shoulder. Atrophy of the thenar muscles may develop in severe cases [2].

Dropping objects (DO) from the hand is a significant complaint among patients with CTS, occurring in up to 50% of cases [3,4]. This symptom may result from a combination of difficulties in sensing the object in the hand, accurately judging grip strength, and maintaining a secure hold on the object. Fine grasping of the hand necessitates a complex interaction between feedforward mechanisms, which control grip strength by anticipating external loads, and feedback mechanisms, which regulate grip strength based on signals from mechanoreceptors [5]. Evidence indicates that the median nerve block at the wrist impairs precision grip, as demonstrated by increases in the safety margin, variations in grip force, and the area of the center of pressure migration. While the nerve block is expected to have minimal impact on feedforward mechanisms, it significantly disrupts sensory feedback and sensorimotor integration [5]. Dysfunction of both afferent sensory and efferent motor components of the median nerve may contribute to the occurrence of DO. It has been reported that the DO symptom is more common in women and the elderly, that these patients have higher Boston Carpal Tunnel Questionnaire (BCTQ) scores and greater severity of neuropathic pain, and that there is impairment of small-fiber conveyed sensation in median and extra-median innervated territories [3,4].

In addition to standard measurements, assessing the segmental conduction velocity (CV) and conduction block (CB) of the median nerve between the palm and wrist provides further support for the diagnosis of CTS [6]. Median CB at the wrist is commonly observed not only in acute/subacute CTS but also in CTS associated with pregnancy [7]. One study reported that, compared to idiopathic CTS, pregnancy-related CTS is characterized by higher rates of CB, along with increased prevalence of diurnal/permanent paraesthesia and more severe symptoms, rather than the classic findings [7]. In this study, the clinical and electrophysiological features of CTS patients with (wDO) and without DO (w/oDO) were compared. Further, the correlation between DO complaint and median CB and median motor CV at the wrist was investigated.

## Materials and methods

Ethical approval and recruitment of subjects

Ethical approval was obtained from the Bezmialem Vakif University Clinical Research Ethics Committee (date: May 2, 2023; application number: 2023/122; decision number: E-54022451-050.05.04-106966). Following the acquisition of both verbal and written consent from the participants, they were included in the study.

The study comprised 201 female subjects aged between 22 and 88, including 57 CTSwOD patients, 78 CTSw/oOD patients, and 66 healthy controls. The diagnosis of CTS was established based on the clinical and neurophysiological criteria outlined by the American Academy of Neurology [8] and the American Association of Electrodiagnostic Medicine [9]. Participants in the CTS groups were recruited from consecutive patients referred to the electromyography (EMG) laboratory for CTS symptoms and confirmed electrophysiologically. Patients aged 18 years or older with electrophysiologically confirmed CTS in at least one hand were included in the CTS groups. CTS patients with anterior horn or root involvement in the C5-T1 segments, peripheral nerve disorders other than CTS, neuromuscular junction diseases, or muscle disorders were excluded from the study. CTS patients who reported DO at least once a week were categorized into the CTSwDO group, while those without such complaints were placed in the CTSw/oDO group. The control group consisted of individuals matched for age distribution with CTS patients, either those referred with a presumptive diagnosis of lower extremity radiculopathy or relatives of hospital staff. Individuals with symptoms indicative of CTS or conditions affecting the peripheral nervous system were excluded from the control group.

Clinical evaluation

Age (years), height (cm), and weight (kg) were recorded for all participants. Body mass index (BMI, weight/height^2^) was calculated for each participant. The presence of diabetes mellitus, hypothyroidism, and other diseases was queried. Incidents of DO and the frequency of such occurrences were recorded. Total weekly occupational hand use time, including writing, phone and computer use, or any tool use, was calculated in hours.

The neurological examination for CTS involved assessing the presence of Tinel’s and Phalen’s signs in the wrist. Sensory disturbances in the median nerve dermatome, weakness in the abductor pollicis brevis (APB) muscle, and thenar atrophy were also evaluated. In the clinical grading of CTS, the classification by the Italian CTS Study Group was used [10]. According to this classification, individuals without CTS symptoms were classified as Stage 0, those with only nocturnal paraesthesia were classified as Stage 1, those with diurnal paraesthesia were classified as Stage 2, those with sensory deficits were classified as Stage 3, those with hypotrophy and/or motor deficits of median innervated thenar muscles were classified as Stage 4, and those with atrophy and/or plegia of median innervated thenar muscles were classified as Stage 5 [10].

The Edinburgh inventory-10 items (EHI) [11], whose Turkish validity and reliability were demonstrated in a study [12] conducted by Atasavun Uysal et al., was utilized. During the questionnaire administration, participants were queried about their hand preferences in 10 different activities (such as writing, brushing teeth, and using scissors). They were instructed to choose from the following options: always left-handed (-10 points), usually left-handed (-5 points), both hands (0 points), usually right-handed (5 points), and always right-handed (10 points). Participants scoring more than 40 points were classified as right-handed, those scoring between 40 and -40 were deemed ambidextrous, and individuals scoring -40 and below were classified as left-handed [11].

To assess the severity and functional impact of symptoms related to CTS, the BCTQ [13] was used, the Turkish version of which was validated by Sezgin et al. [14]. The BCTQ consists of two parts: the Symptom Severity Scale (SSS, 11 questions) and the Functional Status Scale (FSS, 8 questions). The SSS assesses the severity of CTS symptoms, including pain, numbness, tingling, and weakness in the hands and fingers, and asks the participant to rate the severity of their symptoms on a scale of 1 (mild) to 5 (severe). In the FSS, which measures functional limitations caused by CTS, the participants are asked to rate their ability to perform various daily activities, such as writing, grasping objects, and opening jars, on a scale from 1 (no difficulty) to 5 (unable). Higher scores are thought to indicate a more severe CTS [13].

Neurophysiological evaluation

Nerve conduction studies (NCSs) were performed bilaterally in both upper extremities using classical methods described previously [15]. A five-channel EMG device (Natus UltraPro S100 EMG/NCS/EP Neurodiagnostic System, Galway, Ireland) was used. Limb temperatures were carefully maintained between 32°C and 34°C.

In sensory NCSs, bilateral orthodromic stimulation of the median nerve in the second and fourth fingers and the ulnar nerve in the fifth and fourth fingers was performed. Sensory nerve action potential (SNAP) amplitude, latency, and CVs, as well as fourth and second to fifth finger-stimulated median and ulnar SNAP peak latency differences were calculated. Normal values ​​in our laboratory are median SNAP peak latency <3.2 ms, amplitude ≥12 µV, and CV ≥50 m/s; fourthfinger median-ulnar SNAP peak latency difference <0.5; and second finger median-fifth finger ulnar peak latency difference <0.5.

Motor NCSs included examinations of the bilateral median nerve to the APB muscle and the ulnar nerve to the abductor digiti minimi muscle. Compound motor action potential (CMAP) amplitude, distal latency, and CVs were evaluated. Normal values in our laboratory are median CMAP distal latency <4.2 ms, amplitude ≥5 mV, and CV ≥50 m/s. The median nerve was stimulated in the palm (2.5 cm distal to the distal wrist crease), wrist (1.5 cm proximal to the distal wrist crease), and antecubital fossa. Slowing of CV and percentages of decrease in CMAP amplitude at the wrist level were calculated.

The neurophysiological assessment was performed according to the classification suggested by Padua et al. [16]. Those with normal findings on all tests are classified as negative, those with abnormal segmental or comparative tests only are classified as minimal, those with abnormal digit/wrist sensory nerve CV and normal distal motor latency are classified as mild, those with abnormal digit/wrist sensory nerve CV and abnormal distal motor latency are classified as moderate, those with absence of sensory response and abnormal distal motor latency are classified as severe, and those with absence of motor and sensory responses are classified as extreme CTS [16].

Statistical analysis

All analyses were conducted using SPSS version 25.0 (IBM Corp., Armonk, NY, USA). Descriptive statistics for continuous variables were presented as mean ± standard deviation (SD) and as median (minimum-maximum), while categorical variables were given as counts (%). The Kolmogorov-Smirnov test (one-tailed) was employed to assess the normality of the variables. Analysis of variance (ANOVA) was utilized for comparing normally distributed continuous variables, whereas the Kruskal-Wallis test was employed for non-normally distributed data. After Bonferroni correction in post-hoc analyses, pairwise comparisons were performed with the Mann-Whitney U test or independent samples t-test. Pearson’s chi-square test was applied to compare categorical variables. The significance level was set at <0.05.

In the analyses comparing the clinical and electrophysiological findings of CTS, hand dominance was taken into account, as CTS is known to be more pronounced in the dominant hand in individuals without other conditions. It was also considered that the symptom of DO is more likely to result from dysfunction of the dominant hand.

Receiver operating characteristic (ROC) analysis was performed to determine a threshold value capable of distinguishing between CTS and controls based on percent of amplitude decrease in median CMAP at the wrist level or median motor CV at the wrist level. The cut-off value for the percentage decrease in median CMAP amplitude at the wrist level was set at 25. Accordingly, partial CB frequencies were calculated in the CTSwDO and CTSw/oDO groups.

## Results

The mean age of the participants was 56.2 years in the CTSwDO group, 54.2 years in the CTSw/oDO group, and 52.8 years in the control group. There were no significant differences among the groups in terms of age, height, weight, BMI, and handedness (Table 1). There was no difference between wDO and w/oDO CTS patients in terms of the presence of diabetes mellitus, hypothyroidism, or any other accompanying diseases (Table 2).

**Table 1:** Demographic features of the study groups. Categorical variables are presented as numbers (percentage) and numerical variables are presented as mean ± standard deviation (minimum-maximum). *: Kruskal-Wallis H for age, height, and weight; analysis of variance (ANOVA) F for BMI; Pearson’s chi-square for handedness. CTS: carpal tunnel syndrome; wDO: with dropping objects; w/oDO: without dropping objects; N: number of objects; BMI: body mass index; n.s.: non-significant

	CTSwDO (N = 57)	CTSw/oDO (N = 78)	Control (N = 66)	Test statistic*	P-value
Age (year)	56.2 ± 14.1	54.2 ± 12.1	52.8 ± 10.2	1.540	n.s.
	(22-88)	(23-84)	(31-72)		
Height (m)	1.6 ± 0.1	1.6 ± 0.1	1.6 ± 0	2.134	n.s.
	(1.4-1.8)	(1.5-1.8)	(1.4-1.7)		
Weight (kg)	71.8 ± 12.1	73.2 ± 11.6	72.4 ± 10.9	0.103	n.s.
	(48-105)	(48-100)	(44-110)		
BMI (kg/m^2^)	28.3 ± 5.6	28.1 ± 3.9	28 ± 4.3	0.041	n.s.
	(17.7-43.9)	(20.1-37.1)	(19.6-43)		
Handedness					
Right	50 (87.7%)	70 (89.7%)	65 (98.5%)	9.243	n.s.
Left	1 (1.8%)	4 (5.1%)	1 (1.5%)		
Bilateral	6 (10.5%)	4 (5.1%)	0 (0%)		

**Table 2:** Clinical features of carpal tunnel syndrome patients. Categorical variables are presented as numbers (percentage) and numerical variables are presented as mean ± standard deviation (median; minimum-maximum). *: Comparisons between groups were made using Pearson’s chi-square test for categorical variables and the Mann-Whitney U test for numerical variables. CTS: carpal tunnel syndrome; wDO: with dropping objects; w/oDO: without dropping objects; N: number of objects; BCTQ: Boston Carpal Tunnel Syndrome Questionnaire; SSS: symptom severity scale; FSS: functional status scale; n.s.: non-significant

	CTSwDO (N = 57)	CTSw/oDO (N = 78)	Test statistic*	P-value
Diabetes mellitus	17 (30%)	14 (18%)	2.822	n.s.
Hypothyroidism	19 (34%)	29 (37%)	0.150	n.s.
Other disorders	19 (34%)	33 (42%)	0.964	n.s.
Family history for CTS	11 (20%)	15 (19%)	0.012	n.s.
Symptom duration (months)	43 ± 47 (24; 2-192)	38 ± 51 (24; 1-304)	1.709	n.s.
Weekly hand usage (hours)	49 ± 26 (46; 7-114)	45 ± 29 (39.5; 7-128)	1.445	n.s.
BCTQ SSS	30.5 ± 9.4 (31; 12-52)	26.5 ± 8.7 (25.5; 12-51)	6.278	0.012
BCTQ FSS	22.4 ± 8 (23; 8-38)	18.7 ± 8.3 (16; 8-46)	7.408	0.006

In the CTSwDO group, 11 individuals had a family history of CTS, while in the CTSw/oDO group, 15 individuals had such a history. There was no significant difference between the CTSwDO and CTSw/oDO groups in terms of the percentage of median CMAP amplitude decrease at the wrist level and the presence of partial CB. The mean duration of CTS symptoms was 43 months in the CTSwDO group and 38 months in the CTSw/oDO group. The total weekly occupational hand usage time was similar between the CTSwDO and CTSw/oDO groups. In the CTSwDO group, the median BCTQ SSS and FSS scores were significantly higher compared to the CTSw/oDO group (Table 2).

Neurological signs of CTS were significantly more pronounced in the CTSwDO group. These findings are summarized in Table 3. Sensory deficits on both sides were more prominent in the CTSwDO group. Tinel’s/Phalen’s signs and motor deficits on the dominant side were also more pronounced in the CTSwDO group. Consequently, clinical stages were higher in both hands in the CTSwDO group (for the dominant and non-dominant sides, the p-values were 0.036 and 0.023, respectively).

**Table 3:** Clinical examination findings of CTS patients. Variables are presented as numbers (percentages). *: For comparison of the dominant sides of the groups with Pearson’s chi-square test; **: for comparison of the non-dominant sides of the groups with Pearson’s chi-square test. CTS: carpal tunnel syndrome; wDO: with dropping objects; w/oDO: without dropping objects; N: number of objects; D: dominant side; ND: non-dominant side; n.s.: non-significant

	CTSwDO (N = 57)		CTSw/oDO (N = 78)		Test statistic*	P-value*	Test statistic**	P-value**
	D	ND	D	ND				
Tinel’s/Phalen’s sign	39 (74%)	25 (47%)	39 (53%)	31 (42%)	5.683	0.017	0.349	n.s.
Sensory deficit	15 (27%)	13 (24%)	10 (13%)	7 (9%)	3.969	0.046	4.987	0.026
Weakness/Atrophy	25 (46%)	12 (22%)	16 (21%)	9 (12%)	8.551	0.003	2.407	n.s.
Clinical classification								
Stage 0	0	11 (19%)	0	11 (14%)	10.302	0.036	11.361	0.023
Stage 1	3 (5%)	2 (4%)	9 (12%)	9 (12%)				
Stage 2	25 (44%)	25 (44%)	50 (64%)	48 (61%)				
Stage 3	5 (9%)	7 (12%)	4 (5%)	3 (4%)				
Stage 4	24 (42%)	12 (21%)	15 (19%)	7 (9%)				
Stage 5	0	0	0	0				

Median sensory and motor NCS findings are summarized in Table 4. In sensory NCSs, compared to controls, CTS patients had longer median SNAP peak latency, lower SNAP amplitude, and slower CV on both dominant and non-dominant sides. The fourth-finger median-ulnar latency difference and second-to-fifth-finger median-ulnar latency difference were also higher in CTS patients compared to controls. However, there was no significant difference between the CTSwDO and CTSw/oDO groups in terms of median sensory NCSs. The findings of median motor NCSs were different in patients with CTS compared to controls: median CMAP amplitudes were low, latencies were prolonged, and CVs in the wrist and forearm segments were slow. However, there was no significant difference between the CTSwDO and CTSw/oDO groups in terms of median motor NCSs.

**Table 4:** Electrophysiological findings and neurophysiological classification of CTS. Categorical variables are presented as numbers (percentages) and numerical variables are presented as mean ± standard deviation. Comparisons between groups were made using Pearson’s chi-square test for categorical variables and the Kruskal-Willis test for numerical variables. CTS: carpal tunnel syndrome; wDO: with dropping objects; w/oDO: without dropping objects; N: number of objects; D: dominant side; ND: non-dominant side; NCSs: nerve conduction studies; SNAP: sensory nerve action potential; CMAP: compound muscle action potential at the wrist; n.s.: non-significant

	CTSwDO (N = 57)		CTSw/oDO (N = 78)		Control (N = 66)		Dominant		Non-dominant	
	D	ND	D	ND	D	ND	Test statistic	P-value	Test statistic	P-value
Sensory NCSs										
2^nd^ finger SNAP latency (ms)	3.6 ± 0.8	3.9 ± 4.2	3.4 ± 0.7	3.3 ± 0.6	2.5 ± 0.2	2.6 ± 0.2	52.972	<0.001>	33.733	<0.001>
2^nd^ finger SNAP amplitude (µV)	16.6 ± 9.3	18.5 ± 9.9	19 ± 9.5	21.5 ± 11	28.4 ± 8.8	31.3 ± 11.7	28.803	<0.001>	19.235	<0.001>
2^nd^ finger conduction velocity (m/s)	44.1± 8.3	47.2 ± 8.9	44.8 ± 7	46.2 ± 7.8	59 ± 5.7	58.1 ± 4.4	59.832	<0.001>	34.521	<0.001>
4^th^ finger median-ulnar latency difference	1.2 ± 0.9	0.9 ± 0.8	1.2 ± 0.7	0.9 ± 0.6	0.1 ± 0.1	0.1 ± 0.1	61.066	<0.001>	37.427	<0.001>
2^nd^-5^th^ finger median-ulnar latency difference	1.2 ± 0.8	1.5 ± 4.2	1.1 ± 0.6	1 ± 0.5	0.4 ± 0.2	0.3 ± 0.1	47.760	<0.001>	36.683	<0.001>
Motor NCSs										
CMAP latency (ms)	3.9 ± 1.1	3.7 ± 1	3.7 ± 0.9	3.5 ± 0.7	2.9 ± 0.3	2.9 ± 0.3	73.085	0.002	45.205	<0.001>
CMAP amplitude (mV)	8.2 ± 3	8.3 ± 2.6	9.4 ± 3.5	9.2 ± 2.3	11 ± 2.3	11.1 ± 2.2	29.274	<0.001>	33.398	<0.001>
Amplitude loss across the wrist (%)	17.9 ± 19.9	12.9 ± 13	13.5 ± 13.6	11.4 ± 12.8	5.2± 6.2	5.2 ± 6.1	28.555	<0.001>	14.415	0.001
Conduction block, n (%)	13 (23%)	9 (16%)	14 (18%)	12 (15%)	0	0	14.617	0.001	10.460	0.005
Conduction velocity across the wrist (m/s)	29.9 ± 9.5	34.6 ± 11.9	31.5 ± 11	36.5 ± 12.1	55.4 ± 7.4	57.6 ± 6.8	112.315	<0.001>	89.809	<0.001>
Median-Ulnar latency difference	1.6 ± 1.2	1.4 ± 1	1.5 ± 0.9	1.2 ± 0.7	0.5 ± 0.2	0.6 ± 0.3	100.588	<0.001>	45.635	<0.001>
Neurophysiological classification										
Negative		8 (14%)		8 (11%)			6.687	n.s.	2.855	n.s.
Minimal	15 (26%)	11 (19%)	17 (22%)	23 (29%)						
Mild	12 (21%)	14 (25%)	23 (29%)	13 (17%)						
Moderate	20 (35%)	21 (37%)	34 (44%)	29 (37%)						
Severe	10 (18%)	3 (5%)	4 (5%)	5 (6%)						
Extreme	-	-	-	-						

**Figure 1:**
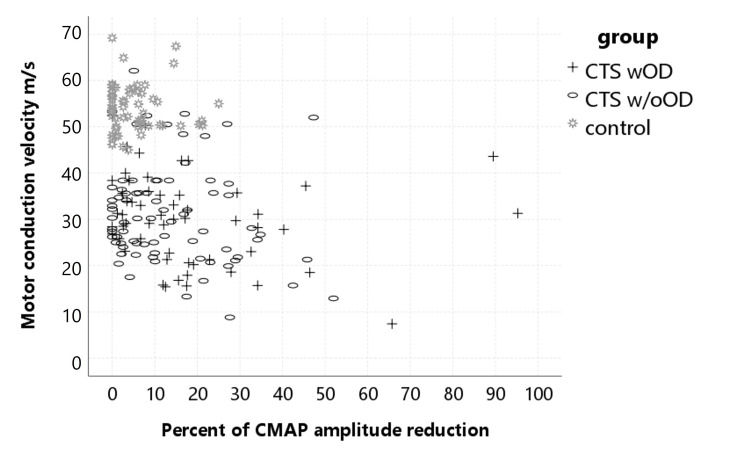
The relationship between median motor conduction velocity and conduction block at the wrist for the dominant side. Normal controls were observed to be concentrated in the upper left part of the graph. No relationship was found between DO and median CV and CB at the wrist level. CMAP: compound muscle action potential; CTS: carpal tunnel syndrome; wDO: with dropping objects; w/oDO: without dropping objects; CV: conduction velocity; CB: conduction block

The percent of median CMAP amplitude reduction at the wrist was correlated with the median motor CV at the wrist on both sides (p-values: dominant side <0.001, non-dominant side <0.001) (Figure 1). There was a correlation between median motor CV in the wrist and weakness in the median innervated thenar muscles (p-values: dominant side = 0.12, non-dominant side <0.001). There was also a correlation between median CB in the wrist and weakness in the median innervated thenar muscles (p-values: dominant side = 0.15, non-dominant side = 0.15).

**Figure 2:**
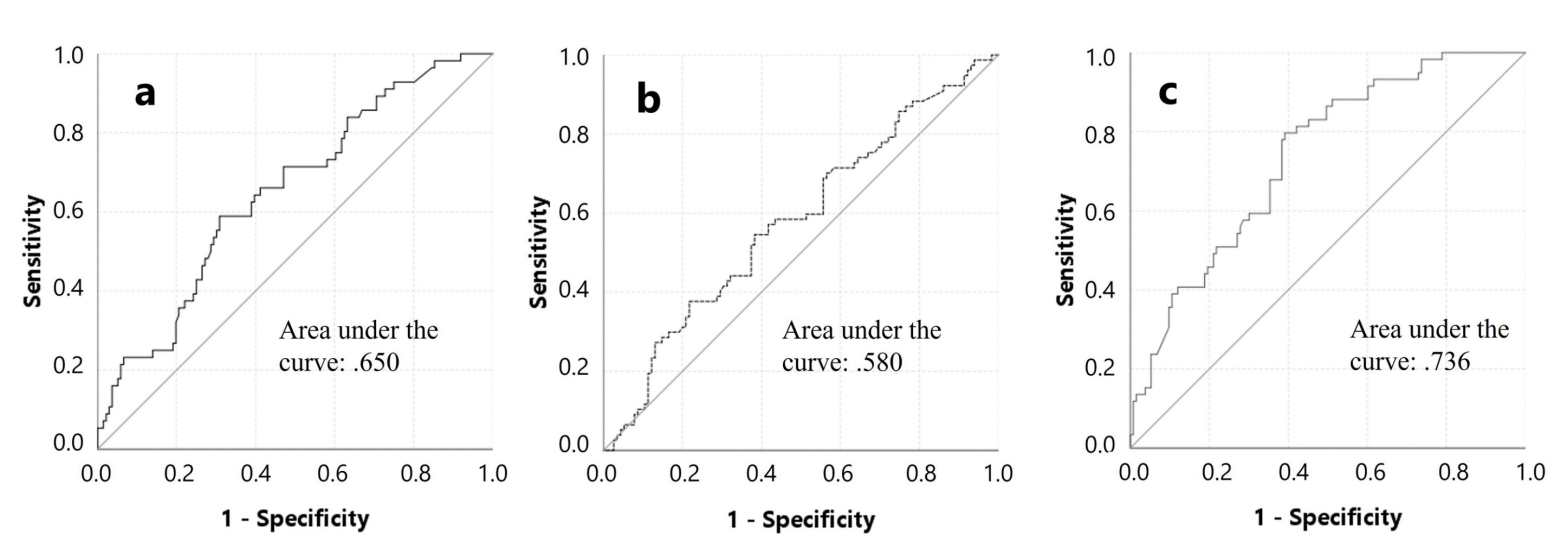
ROC curve for reduction percent in median CMAP amplitude at the wrist level for the dominant side. It was plotted to find a limit value that could distinguish the CTSwDO group (a), CTSw/oDO group (b), and normal group (c) from others. When the percentage of amplitude loss at the wrist is more than 25, it can be said that the person is in the CTSw/oDO group (a) with 23% sensitivity and 88% specificity and in the CTSw/oDO group (b) with 20% sensitivity and 88% specificity. On the other hand, when the percentage of amplitude loss is 25 or less, it can be said that the person is normal with 100% sensitivity and 21% specificity (c). CMAP: compound muscle action potential; CTS: carpal tunnel syndrome; wDO: with dropping objects; w/oDO: without dropping objects; ROC: receiver operating characteristic

**Figure 3:**
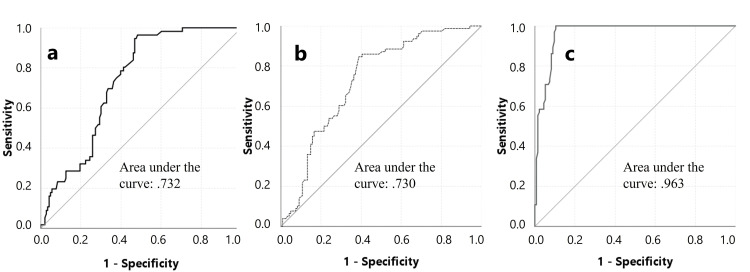
ROC curve for median motor CV at the wrist level for the dominant side. It was plotted to find a limit value that could distinguish the CTSwDO group (a), CTSw/oDO group (b), and normal group (c) from others. If the CV is 45 m/s or slower, it can be said that the person is in the CTSwDO group with 95% sensitivity and 52% specificity, and the person is in the CTSw/oDO group with 86% sensitivity and 45% specificity. If the CV is faster than 45 m/s, the person can be said to be in the normal group with 100% sensitivity and 90% specificity. CTS: carpal tunnel syndrome; wDO: with dropping objects; w/oDO: without dropping objects; ROC: receiver operating characteristic; CV: conduction velocity

There was no significant difference between the CTSwDO and CTSw/oDO groups in terms of the percentage of median CMAP amplitude decrease at the wrist level (Figure 2) and the presence of partial CB as well as median motor CV at the wrist level (Figure 3). There was no significant difference between the groups in terms of ulnar sensory and motor NCS findings.

## Discussion

While paraesthesia and pain in the first three fingers of the hand that worsen at night are the most common symptoms of CTS, clumsiness in the hand is also a common and bothersome symptom. A study that evaluated the DO symptom for the first time in patients with CTS reported that this symptom occurred in approximately half of the patients affected by CTS, while only 3% of the control group reported DO [3]. In this study, which investigated the relationship between frequent DO and the clinical and electrophysiological severity of CTS, we found that DO may be a symptom of clinical severity in CTS, as previously reported [3,4]. In the CTSwDO group, when compared to the CTSw/oDO group, BCTQ SSS and FSS scores were significantly higher, the incidence of sensory deficits was more pronounced, and a greater number of hands, both dominant and non-dominant, were classified as grade 3 or higher in the clinical CTS classification. However, no neurophysiological parameter that could be associated with dropping an object has been identified.

The presence of paraesthesia or pain in at least two of the first four fingers along with the presence of one of that are female gender, worsening of symptoms at night or on awakening, BMI ≥30 kg/m^2^, thenar atrophy, or other symptoms (Tinel’s, Phalen’s, or reverse Phalen’s signs) were found to be the most valuable indicators in the clinical diagnosis of CTS [17]. The diagnosis of CTS is primarily based on clinical signs and symptoms. EMG is very useful in determining the type (axonal, demyelinating) and the degree of median nerve involvement and is an important neurophysiological test in the diagnosis and follow-up of CTS [10,16]. However, the use of clinical criteria in diagnosing CTS generally leads to a higher estimate compared to using electrophysiological criteria [2]. When only clinical presentation is used to define the syndrome, the variability in prevalence findings arises from the choice between broad criteria (such as history or Phalen’s test) and strict criteria (such as sensory or motor deficits) [2]. In this study, the clinical diagnostic criteria established by the Italian CTS Study Group were employed [10]. Given that only patients with electrophysiologically confirmed CTS were included, cases classified as very mild were not included in the study.

Various scales are employed to evaluate the disability caused by CTS, including the BCTQ, the Disabilities of the Arm, Shoulder, and Hand, and the Michigan Hand Outcomes Questionnaire [18,19]. Additionally, neuropathic pain associated with CTS is assessed using scales such as the Douleur Neuropathique 4 questions (DN4) and the Numeric Rating Scale for pain [20,21]. In the present study, the BCTQ was utilized to assess both the severity of CTS symptoms and their impact on daily functioning. The BCTQ is a highly reliable, patient-oriented instrument that offers valuable insights into the patient’s perspective on CTS [18]. In our previous research, which explored the relationship between DO and CTS symptoms among dentists under 40 years of age, we identified a significant correlation between BCTQ scores and the frequency of DO and suggested that accidental DO could be a functional indicator of mild CTS, even in the absence of electrophysiological abnormalities [22]. In this study, BCTQ scores (SSS and FSS) were observed to be significantly higher in the CTSwDO group compared to the CTSw/oDO group. This finding aligns with previously reported results [3,4,23].

Neurological examination findings including Tinel’s/Phalen’s sign, sensory deficit, and weakness/atrophy in the median innervated thenar muscles were more evident in the CTSwDO group and the dominant hand in both CTS groups. The most common positive finding was the presence of Tinel’s/Phalen’s sign with an occurrence rate of 42-74%. It was consistent with relevant literature [2]. The reported sensitivity ranges from 42% to 85% for Phalen’s maneuver and from 38% to 100% for Tinel’s test. The specificity ranges from 54% to 98% for Phalen’s maneuver and from 55% to 100% for Tinel’s test [2].

In this study, distinguishing CTS patients from controls, the cutoff value for median motor CB at the wrist level was identified as more than a 25% amplitude decrease and a CV slower than 45 m/s. However, no cutoff value for these two parameters (median motor CB and CV slowing at wrist level) could be determined to distinguish between CTSwDO and CTSw/oDO. A strong correlation was found between the decrease in the median CMAP amplitude at the wrist, the slowdown in the median CV, and weakness or atrophy in the wrist. Conversely, no relationship was found between hand clumsiness and any neurophysiological findings. A study by Kim et al. showed that, in patients with osteoarthritis and CTS, the amplitude of the median CMAP is a strong predictor of hand strength, and that the velocity of the median CMAP and the amplitude of the median SNAP are strong predictors of hand dexterity [24]. It has been shown that axonal hyperpolarization resulting from voluntary contraction has little effect on the security of impulse conduction in motor axons in patients with mild-to-moderate CTS [25]. Numerous morphological and physiological factors can cause conduction slowing independent of demyelination. These factors include axonal tapering, remyelination with shorter internodes, nodal intussusception, axonal depolarization, axonal hyperpolarization, Na^+^ channel blockade, and cooling. The classical worsening of CTS symptoms at night may be due to ischemia-induced axonal depolarization block. These symptoms are characteristically relieved by hand movement, which presumably restores function by alleviating the ischemia and thereby the depolarization block [25].

The incongruity between motor symptoms and neurophysiological findings has been previously documented. A significant correlation has been established between the severity of hand weakness and clumsiness symptoms and the intensity of sensory symptoms, including pain, numbness, and tingling [23]. Tamburin et al. identified a correlation between motor symptoms in the hand and the warm threshold detected by Quantitative Sensory Testing that offers a precise evaluation and quantification of sensory function by determining detection thresholds. They proposed that “noxious C-fiber input may reduce motor cortex excitability and inhibit motor cortex oscillatory activity,” suggesting that central nervous system motor inhibition due to C-fiber dysfunction might underlie hand weakness and clumsiness in CTS. In the study by Yen et al., which investigated the impact of sensory findings on motor control, it was shown that individuals with low SNAP amplitude exhibited significantly diminished hand sensitivity and reduced efficiency in force adjustment [26]. Moreover, a recent study by Isoardo et al. identified thermal hypoesthesia in median and extra-median regions as predictors of DO in CTS [4]. They further reported that the presence of neuropathic pain, as defined by DN4, doubled the likelihood of DO from the hands [4].

This study has several limitations. One notable limitation is the inability to assess the strength of the APB muscle using a hand dynamometer, which might have provided more accurate measurements compared to clinical examination alone. Additional limitations include the lack of tests to evaluate manual dexterity, allodynia, and pain. Furthermore, the evaluation of BCTQ subscores was not conducted. Moreover, the study’s sample size was relatively small and limited by the lack of a multicenter approach.

## Conclusions

DO from the hand was a symptom commonly observed in our patients with CTS, as previously reported. This symptom has been found to have a strong correlation with the BCTQ SSS and FSS scores, as well as with the clinical grading of CTS. However, it cannot be directly attributed to the effects on motor nerve fibers. No significant relationship was identified between this symptom and median motor CB, CV at the wrist, or other neurophysiological parameters.
